# SARS-CoV-2–Related Adaptation Mechanisms of Rehabilitation Clinics Affecting Patient-Centered Care: Qualitative Study of Online Patient Reports

**DOI:** 10.2196/39512

**Published:** 2023-04-13

**Authors:** Lukas Kühn, Lara Lindert, Paulina Kuper, Kyung-Eun Anna Choi

**Affiliations:** 1 Center for Health Services Research Brandenburg Medical School Rüdersdorf bei Berlin Germany; 2 Medical Image Analysis & Artificial Intelligence Research Centre Health Services Research Group Danube Private University Krems-Stein Austria

**Keywords:** patient-led care, patient autonomy, patient report, satisfaction, pandemic, coronavirus, inpatient, health care delivery, service delivery, rehabilitation, internet, web-based, reviews, complaint, rating, COVID-19

## Abstract

**Background:**

The SARS-CoV-2 pandemic impacted access to inpatient rehabilitation services. At the current state of research, it is unclear to what extent the adaptation of rehabilitation services to infection-protective standards affected patient-centered care in Germany.

**Objective:**

The aim of this study was to determine the most relevant aspects of patient-centered care for patients in inpatient rehabilitation clinics under early phase pandemic conditions.

**Methods:**

A deductive-inductive framework analysis of online patient reports posted on a leading German hospital rating website, Klinikbewertungen (Clinic Reviews), was performed. This website is a third-party, patient-centered commercial platform that operates independently of governmental entities. Following a theoretical sampling approach, online reports of rehabilitation stays in two federal states of Germany (Brandenburg and Saarland) uploaded between March 2020 and September 2021 were included. Independent of medical specialty groups, all reports were included. Keywords addressing framework domains were analyzed descriptively.

**Results:**

In total, 649 online reports reflecting inpatient rehabilitation services of 31 clinics (Brandenburg, n=23; Saarland, n=8) were analyzed. Keywords addressing the care environment were most frequently reported (59.9%), followed by staff prerequisites (33.0%), patient-centered processes (4.5%), and expected outcomes (2.6%). Qualitative in-depth analysis revealed SARS-CoV-2–related reports to be associated with domains of patient-centered processes and staff prerequisites. Discontinuous communication of infection protection standards was perceived to threaten patient autonomy. This was amplified by a tangible gratification crisis of medical staff. Established and emotional supportive relationships to clinicians and peer groups offered the potential to mitigate the adverse effects of infection protection standards.

**Conclusions:**

Patients predominantly reported feedback associated with the care environment. SARS-CoV-2–related reports were strongly affected by increased staff workloads as well as patient-centered processes addressing discontinuous communication and organizationally demanding implementation of infection protection standards, which were perceived to threaten patient autonomy. Peer relationships formed during inpatient rehabilitation had the potential to mitigate these mechanisms.

## Introduction

In modern health systems, the relevance of patient-centered care (PCC) continues to progress as it is associated with improved patient satisfaction, self-management, and perceived quality of care [1,2]. The SARS-CoV-2 pandemic impacted endeavors of PCC on inpatient rehabilitation services (IRS). In Europe, an estimated range of 1.3 to 2.2 million patients were required to pause their rehabilitation program in March 2020 [3]. Since then, inpatient rehabilitation clinics have learned to adapt their services to incorporate infection-protective standards while trying to equally uphold the quality of care [4,5]. For instance, geriatric rehabilitation clinics faced capacity shortages; consistent admission delays to rehabilitation services; restricted access to therapists, social workers, or pharmacists; and impacted process parameters such as reduced interprofessional team meetings, structured discharge planning, or shared decision-making efforts [6]. However, it is evident that the extent of adaptation mechanisms varied internationally [7]. Compared to other high-income countries, Germany opted for lockdowns early on, accepting high socioeconomic costs to protect society [7]. It is therefore reasonable that the rigorous implementation of the German infection-protection policy not only affected societal lives but also general health care such as IRS. At present, there is insufficient evidence of the extent to which these adjustments influenced PCC in German inpatient rehabilitation clinics. As a growing number of patients use web-based tools to provide feedback on their experience during medical service claims [8], the aim of this study was to systematically analyze patient experience reports of a clinic rating website in Germany considering patients’ perspectives on how SARS-CoV-2–related adaptation mechanisms affected PCC during inpatient rehabilitation.

In Germany, approximately 85% of medical rehabilitation services are provided in inpatient care settings [9]. The central objective of German inpatient rehabilitation is to reduce the effects of disabled conditions on social inclusion so as to prevent occupational incapacity or the need for long-term care [10]. In most countries, the initiation of rehabilitation follows a serious medical event and/or a major surgical intervention. However, in Germany, 75% of rehabilitation services target preventive services addressing chronic diseases and disabilities with a progressive course [11].

Due to a historically grown separation of acute care and medical rehabilitation, the German system faces declining trends of rehabilitation claims as patients are self-responsible to initiate application processes to IRS and intersection communication among health care sectors is fragmented [9]. Since patients can mostly choose the facilities of rehabilitations themselves, there has traditionally been a culture of competitive advertising, not only with regard to medical equipment but also in response to PCC components. The SARS-CoV-2 pandemic intensified this situation as the number of medical rehabilitation requests decreased by 14.5% in the first year of the pandemic [12].

Looking at other inpatient care settings, Andersson et al [13] investigated adaptation mechanisms of critical care nurses affecting person-centered care structures. The interviewed nurses felt unprepared to deal with conditions associated with the SARS-CoV-2 pandemic. Considering PCC processes, they experienced limited patient communication, and evaluated care to be impersonal and driven by routines. Overall, they sensed patients to be objectified as they perceived a main focus on diagnosing SARS-CoV-2 in new arrivals. Ward managers of a university hospital in Denmark additionally reported influences of the pandemic on person-centered leadership endeavors: holding an intersection position between the clinic management and the nursing staff, they experienced a lack of appropriate involvement in decision-making structures and acknowledgment of individual perspectives [14]. The authors argued that this top-down management approach negatively affected the engagement of ward managers, potentially affecting the quality of care.

Considering limited or delayed admission to IRS, changed process parameters, reduced availability of services, and nontransparent longitudinal leadership structures, it is unclear to what extent these adaptations affected PCC during rehabilitation in Germany. Moreover, an appropriate inclusion of patient perspectives is currently pending. Thus, the particular interest of this study was to evaluate which aspects of PCC were important for IRS recipients during the early phase of the SARS-CoV-2 pandemic in Germany. In that regard, the following research question motivated this study:

Which aspects of PCC are relevant for patients in inpatient rehabilitation clinics and how do they evaluate these aspects to be achieved under conditions of the SARS-CoV-2 pandemic?

By identifying SARS-CoV-2–related aspects affecting PCC in inpatient rehabilitation, the research team aimed at informing rehabilitation clinics to not only become resilient health care organizations but also to meet patient needs in highly demanding and exceptional circumstances of the future. Despite stating palpable organizational interests, this ambition also reflects a moral attitude being of central relevance to any health care organization.

## Methods

### Theoretical Framework

**Figure 1:**
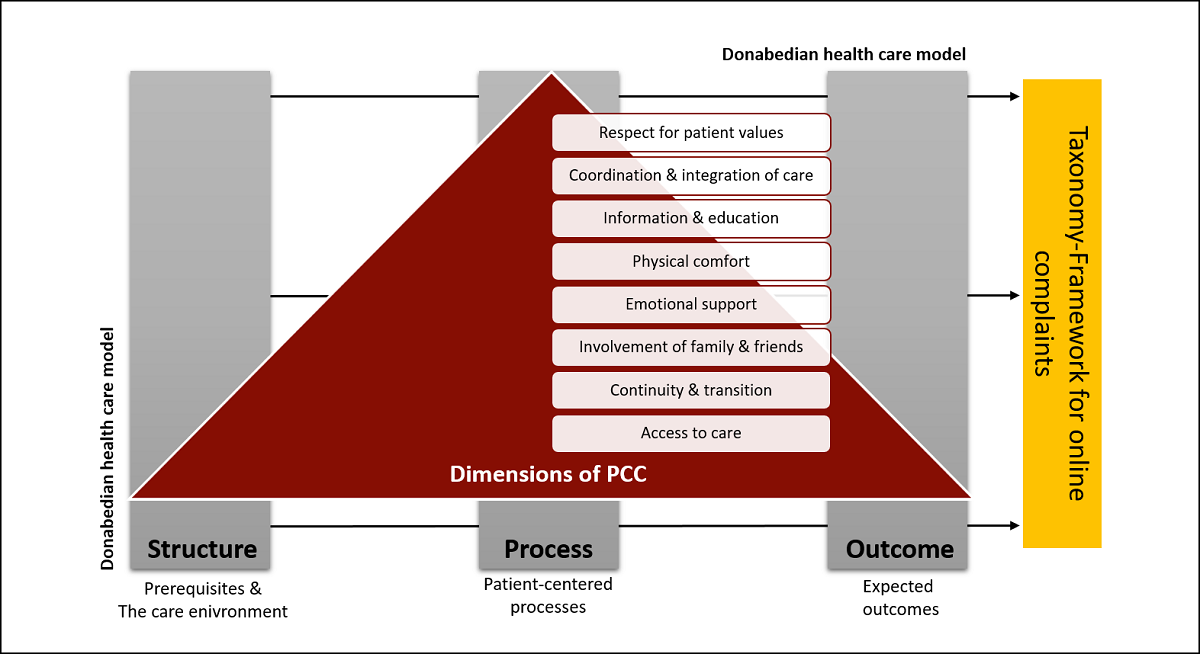
Theoretical framework introduced by Liu et al [15]. PCC: patient-centered care.

In this qualitative analysis, a deductive-inductive framework approach was used. The applied framework was developed by Liu et al [15] aiming at categorizing online patient complaints into a PCC perspective. The development was guided by the best fit framework synthesis technique [16] and tailored accepted PCC-framework models to the data source of online patient complaints. According to Coulter [17], PCC is a form of care that meets and responds to patients’ wants, needs, and preferences, and is prevalent where patients are autonomous and able to decide for themselves. The main dimensions affecting PCC are: (1) respect for patient values, preferences, and expressed needs; (2) coordination and integration of care; (3) information and education; (4) physical comfort; (5) emotional support and alleviation of fear and anxiety; (6) involvement of family and friends; (7) continuity and transition; and (8) access to care [15,18]. Grounded by this concept, Liu et al [15] integrated the dimensions of PCC into the Donabedian structure-process-outcome model containing the following four constructs: (1) prerequisites, (2) the care environment, (3) patient-centered processes, and (4) expected outcomes [19]. In a second step, the taxonomy was tested by assigning themes derived from the quantitatively selected patient online complaints data into the framework. Figure 1 illustrates the applied framework.

### Sample Selection and Data Source

The sample of online posted patient reports was guided by a theoretical selection process. Reports on hospital stays posted on the most commonly used German hospital rating website Klinikbewertungen (Clinic Reviews) [20] were included if written between March 2020 and September 2021. This time period represents the phase in which initial hygiene protection standards (eg, mandatory masks, distance regulations, test obligation) were implemented and maintained across German rehabilitation clinics [21]. The selected hospital rating website is a third-party, patient-centered commercial platform that operates independently of governmental entities. Whether hospitals encourage patients to rate their hospital stay on this platform cannot be answered with certainty.

Given that this was an exploratory study, patient reports were included regardless of their medical indications. To contrast results, reports of IRS were included if referred rehabilitation clinics were located in the federal states of Brandenburg and Saarland, as these states demonstrated the highest and lowest decrease of applications for IRS provided by the Federal German Pension Fund, respectively (Saarland=58.3%, Brandenburg=23.9%) [12]. The federal state of Saarland is located in southwest Germany with a population density of 186 citizens/km^2^ [22]. Brandenburg is located in northeast Germany with a density of 85 residents/km^2^ [22]. Despite infrastructural differences, differences in IRS application rates may imply different coping strategies of resident patients of the respective states or may further reflect different political strategies at the federal policy level.

### Ethical Considerations

In this study, open-access online patient reports were used. Therefore, no ethical approval was required. However, we carefully anonymized all cited reports in the manuscript to avoid a linkage of patients’ user names of the hospital rating websites with referenced citations.

### Data Extraction

Data on patient reports were extracted using a web-scraping technique based on the computing package “Rvest” of the R Project for Statistical Computing [23]. The web-scraping code of included data is provided in Multimedia Appendix 1. Scraped data were transferred to Microsoft Excel (Redmond, USA) and imported into the qualitative analysis software MAXQDA (Berlin, Germany).

### Data Analysis

#### Quantitative Analysis

The total and relative numbers of included rehabilitation clinics and their representing specialties were calculated. Keywords representing domains and categories of the applied framework were analyzed descriptively by reporting absolute and relative frequencies. Additionally, geographic differences in keyword distributions between the included federal states of Brandenburg and Saarland were tested by the *χ^2^* distribution with a set significance threshold of *P*≤.05. State-specific average word count differences per online report were tested for significance by applying *t*-test statistics. Quantitative text data management was ensured by MAXQDA, which offers an analytical software for qualitative data management. Statistical analysis of text data was conducted via Microsoft Excel.

#### Qualitative Analysis

A deductive-inductive framework analysis was performed. Two researchers (LK and LL) independently pilot-coded patient reports of two rehabilitation clinics (n=72 online reports), which were randomly selected. After discussing discrepancies and achieving consensus, one researcher (LK) coded the pending data. The coding tree comprised 4 domains, 8 categories, and 25 subcategories of PCC reflecting the introduced framework of Liu et al [15]. Additional themes were coded inductively. By following this approach, the credibility of qualitative data analysis was guaranteed by investigator and theory triangulation.

Anchor quotes representing key findings of the qualitative analysis were preselected and translated into the English language by one researcher (LK). The selection and translation were cross-validated for representativeness and consistency by a second researcher (AC). Data management was provided by using MAXQDA. Data reporting was guided by the COREQ (consolidated criteria for reporting qualitative research) checklist [24] and is provided in Multimedia Appendix 2. The research group has occupational experience in health services research (AC, LL, LK, PK), psychology (AC, PK), physiotherapy (LK), and rehabilitation science (LL).

## Results

In total, 43 rehabilitation clinics are located in the federal states of Brandenburg and Saarland, 31 of which are listed on the investigated hospital rating website. Within clinics, 11 medical specialty groups are settled with orthopedic (n=14, 23%), internal medicine (n=17, 28%), and psychiatric/psychosomatic (n=13, 22%) facilities, representing the most frequent specialty groups. During the targeted time period, a sample of 659 posted patient reports was identified. As 10 reports were recognizably related to rehabilitation stays prior to the SARS-CoV-2 pandemic, the final included sample size was 649 reports. State-specific sample characteristics are summarized in Table 1.

**Figure 2:**
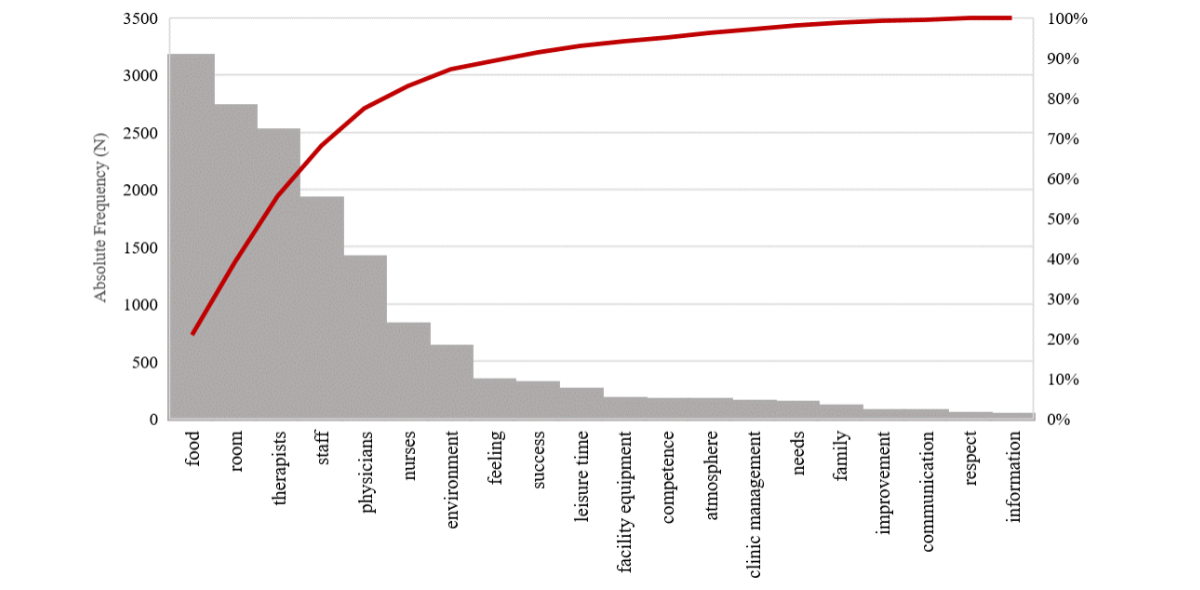
Frequencies of keywords addressing PCC domains. PCC: patient-centered care.

Among the total of 15,125 keywords across federal states and medical specialty groups, keywords relating to food (n=3160, 20.89%) and room amenities (n=2721, 17.99%) were predominantly reported. This was followed by keywords associated with medical and administrative specialty groups, with therapeutic professions being the most commonly cited, including therapists (n=2513, 16.61%), staff (n=1915, 12.66%), physicians (n=1402, 9.27%), and nurses (n=820, 5.42%). Keywords relating to outcome expectancies and information provision were numerically the least represented categories (improvement: n=67, 0.44%; communication: n=66, 0.44%; information: n=30, 0.20%). The cumulative distribution of included keywords is additionally illustrated in Figure 2.

Comparing the average word count per online report, no significant differences across states were identified (Brandenburg, n=140.1 words; Saarland, n=148.3 words; *P*=.75). According to differences of keyword distributions across the federal states of Brandenburg and Saarland, significant differences were identified in PCC domains of prerequisites as well as the care environment. Within the domain of prerequisites, keyword distributions addressing medical specialty groups of therapists, nurses, and physicians significantly differed between states. Within the domain of the care environment, keyword distributions addressing food, room amenities, the environment, and administrative staff significantly differed between states. Within domains of patient-centered processes as well as expected outcomes, no significant differences of distributions were observed. Detailed information on observed keyword frequencies within the included patient reports is provided in Table 2.

**Table 1:** Sample characteristics of online patient reports.

Characteristics	Brandenburg	Saarland	Total
Rehabilitation clinics, n (%)	27 (64)	15 (36)	42 (100)
Rehabilitation clinics listed online in the rating portal, n (%)	23 (74)	8 (26)	31 (100)
Represented specialty groups, n (%)	11 (100)	8 (73)	11 (100)
Patient reports, n (range)	478 (2-85)	181 (8-64)	659 (2-85)

**Table 2:** Differences of keyword frequencies across two federal states in Germany.

Patient-centered care domain			Brandenburg (n=11,234), n (%)		Saarland (n=3891), n (%)		Total (n=15,125), n (%)		*P* value
**Prerequisites**									
	Competence	121 (1.1)		43 (1.1)		164 (1.1)		.88	
	Therapists	1757 (15.6)		756 (19.4)		2513 (16.6)		<.001	
	Nurses	751 (6.7)		69 (1.8)		820 (5.4)		<.001	
	Physicians	1074 (9.6)		328 (8.4)		1402 (9.3)		.03	
**Patient-centered processes**									
	Respect	26 (0.2)		10 (0.3)		36 (0.2)		.78	
	Family	78 (0.7)		23 (0.6)		101 (0.7)		.50	
	Feeling	234 (2.1)		98 (2.6)		332 (2.2)		.11	
	Needs	100 (0.9)		39 (1.0)		139 (0.9)		.53	
	Communication	48 (0.4)		18 (0.5)		66 (0.4)		.77	
	Information	18 (0.2)		12 (0.3)		30 (0.19)		.07	
**The care environment**									
	Food	2292 (20.4)		868 (22.3)		3160 (20.9)		.01	
	Room	1881 (16.7)		840 (21.6)		2721 (18.0)		<.001	
	Environment	527 (4.7)		101 (2.6)		628 (4.2)		<.001	
	Leisure time	188 (1.7)		60 (1.5)		248 (1.6)		.58	
	Staff	1498 (13.3)		417 (10.7)		1915 (12.7)		.001	
	Clinic management	101 (0.9)		40 (1.0)		141 (0.9)		.47	
	Atmosphere	126 (1.1)		37 (1.0)		163 (1.1)		.37	
	Facility equipment	126 (1.1)		41 (1.1)		167 (1.1)		.72	
**Expected outcomes**									
	Success	242 (2.2)		67 (1.7)		309 (2.0)		.10	
	Improvement	45 (0.4)		22 (0.6)		67 (0.4)		.18	

The following sections provide a qualitative in depth-analysis of online composed patient reports guided by the domains of the introduced PCC framework.

### Prerequisites

Within this domain, attributes of the patient-centered professional emerged to be of major significance for patients utilizing the referred hospital rating website. As a main result, patients felt a decreased sensitivity and a lack of empathy in interpersonal interactions between themselves and the medical staff. They perceived some physicians and therapists to exploit the naturally prevalent hierarchy among them and interpreted this patronizing human interaction as an expression of an imminent gratification crisis.

If you complain, they shoot back immediately and you have to shut up and pull yourself together […]. I have also noticed that some doctors and therapists think that patients are inferior and are here to be re-educated. In general, it seems to me that everyone has lost the desire to do their job. [...] Of course: there are exceptions

Orthopedic-psychosomatic rehabilitation clinic, Saarland

### Patient-Centered Processes

Among reports, a discontinuous communication of curfew legislation supported a perceived sense of disempowerment. This was further endorsed by hygiene rules, which were rated to be arbitrary as they noticeably differed across rehabilitation clinics and impacted the perceived autonomy of a relevant number of patients. Complaints addressing a decrease of autonomy were particularly prevalent in psychiatric facilities. However, a majority of patients rated existing hygiene rules to be appropriate.

In general, a prison character arises from the incapacitating, uncomprehending habitus of some therapists. Those to whom self-determination is an important value will not be happy here.

Psychiatric rehabilitation clinic, Saarland

Referring to care continuity, intersectoral care was not always maintained. Patients criticized a lack of involvement of their family physician or their psychologist in charge. Apparently, this was reflected by the fact that diagnostic reports and treatment plans of ambulatory care were frequently not taken into account during IRS. Conversely, results of IRS were not transferred to the ambulatory health care practitioner. Moreover, patients reported to have limited access to structured ambulatory follow-up rehabilitation programs as responsible social workers were hard to reach. Despite stated constraints, established clinician-patient relationships had the potential to mitigate adverse effects of the pandemic on IRS as patients valued empathetic, personal contact.

I was in [rehabilitation clinic] for four weeks for rehab of my cervical spine and diabetes. Even the 12-hour quarantine (it was Corona time) flew by, as even during that time everyone from the kind staff and nurses cared about my well-being.

Orthopedic-diabetic rehabilitation clinic, Brandenburg

### The Care Environment

Availability of therapeutic and nursing care was mainly attributed to the care environment. Patients reported having limited access to therapeutic and nursing procedures. In some cases, this limited availability of care led to a termination of the inpatient rehabilitation stay.

In 3 months I was showered three times. When I asked for a shower as an incomplete paraplegic, I was told maybe tomorrow due to sparse staff availability. Sorry, but what? […] Even after talking to doctors, nothing has really changed. All in all, I have mixed feelings and it is very important not to blame everything on Corona.

Oncological rehabilitation clinic, Brandenburg

Despite availability issues, it became apparent that hygiene legislations were more likely to be accepted if they were easily integrated into organizational routines. This was also seen as having the advantage to create a more familiar environment as, for instance, therapeutic care groups decreased in size. One factor not directly attributable to the pandemic was the available food, which was perceived to be inconsistent with nutrition education events offered during rehabilitation.

Due to the Corona pandemic, procedures were changed which wasn’t only bad: For instance, I perceived the cutting of the reference group actually very pleasant and more personal. I perceived the sessions to be more intense and individual. Perhaps, it should be considered whether this can be maintained after the pandemic.

Psychiatric rehabilitation clinic, Brandenburg

### Expected Outcomes

In general, the domain of expected outcomes was of minor significance for patients in German rehabilitation clinics. However, it became apparent that a distinct communication of patient-relevant outcomes and their respective change after rehabilitation was positively associated with patient satisfaction. The communication of changes in outcomes seemed to be more straightforward to be implemented in somatic care facilities. Furthermore, available emotional support during IRS was perceived to facilitate the individual healing process by having a direct impact on activating self-efficacy and self-management potential.

I want to compliment the care provided by doctors and therapists. I arrived here with severe swelling and effusion in the knee and leave the rehab with great mobility and stability in my joint (70° on arrival 115° on departure).

Orthopedic rehabilitation clinic, Saarland

I arrived as a diabetic with overweight, having taken medication for three years, including for high blood pressure. The holistic care of the staff has resulted in, me losing 12 kilograms in four weeks and I am now medication free. My long-term blood sugar now is 5.9 and I’m coming from over 8.

Orthopedic-diabetic rehabilitation clinic, Brandenburg

### Peer Relationship

The domain of “peer relationship” inductively emerged during the process of analysis. Empowering peer-to-peer relationships was valued to have the potential to mitigate adverse effects of the pandemic on IRS. Thus, some patients reported that their stay remains unforgotten mainly due to their peers, who compensated for negative inconveniences. Patients also appealed to the personal responsibility of their peers. In their understanding, only active engagement allows expectations of rehabilitation success.

A rehab is not a vacation, your own participation is expected and necessary- success depends on you and your attitude toward rehab and your own illness; a rehab facility is not a hotel with many stars…

Psychiatric rehabilitation clinic, Brandenburg

The first week was shaped by uncertainty of the unknown, but the great people I met supported me to deal with the problems that arose. Usually, we would meet for lunch to tell each other what had happened during the day. Many times, we were just listeners when a colleague of ours was feeling bad.

Psychiatric rehabilitation clinic, Brandenburg

A comprehensive summary of online reported patient experiences addressing PCC domains and attributes is provided in Table 3.

**Table 3:** Key statements from patient reports related to inpatient rehabilitation service (IRS) during the SARS-CoV-2 pandemic.

Domain and attributes			Experiences
Prerequisites^a^: attributes of the patient-centered professional			Perceived gratification crisis of medical staff
**Patient-centered processes^a^**			
	Patient as a source of control	Discontinuous communication of curfew legislation creates a sense of disempowerment	
	Patient autonomy	To maintain hygiene legislation, the patient decision-making autonomy is restricted, which is frequently perceived as arbitrariness	
	Family and friends as supported caregivers	Lacking leisure time activities for companions	
	Transition and continuity of care	Intersectoral care continuity is not always maintained	
	Care based on a continuous healing relationship	Members of the nursing and therapy professions are perceived to be more trustworthy than physicians	
	Clinician-patient relationship	Established clinician-patient relationships have the potential to mitigate adverse effects of the pandemic on IRS	
**The care environment^a^**			
	Availability	Availability of therapeutic and nursing services was in part severely limited	
	Supportive organizational system	Acceptance of hygiene legislations increases if they can easily be integrated into organizational routines	
	Therapeutic environment	Adapted routines create a personal, familiar environment; nutritional theory and lived practice are inconsistent	
**Expected outcomes^a^**			
	Physical comfort	Distinct communication of therapeutic outcomes supports patient satisfaction	
	Emotional support; alleviation of anxiety	Emotional support promotes the healing process and self-management	
**Peer relationship^b^**			
	Peer as a supported person of trust	Established peer-to-peer relationships have the potential to mitigate adverse effects of the pandemic on IRS	
	A call for personal responsibility	IRS is to be appraised in addition to personal responsibility	

^a^Deductive domain.

^b^Inductive domain.

## Discussion

### Principal Findings

For patients receiving IRS, aspects of the care environment, staff prerequisites, and patient-centered processes were predominantly relevant to evaluate their inpatient stay. SARS-CoV-2–related adaptation mechanisms affecting these domains comprised discontinuous communication and elaborate implementation of infection protection standards, which were perceived to threaten the personal autonomy of action. These mechanisms were amplified by tangible gratification crises of medical staff. However, the prevalence of established and emotional supportive relationships to clinicians and peer groups provided the potential to mitigate the adverse effects of hygiene protection standards on IRS. Moreover, a distinct communication of therapeutic outcome variation seemed to support patient satisfaction. These insights provide the opportunity to develop informed strategies fostering resilient organizations that sustainably embody PCC within the setting of rehabilitative care.

### Comparison to Prior Work

Our findings are partly in line with those of Liu et al [15] who demonstrated country-specific differences in patient complaint behaviors. Although British and Canadian reviewers tend to complain about staff prerequisites, Germans are more likely to criticize the care environment and patient-centered processes [25]. Considering the context of inpatient rehabilitation clinics, Sander et al [26] conducted an initial examination of web-based patient reports to investigate determinants associated with recommending inpatient rehabilitation clinics, and identified perceived therapy successes as well as process of care parameters to be associated with clinic recommendations. Although aspects of patient-centered processes and expected outcomes were quantitatively subordinate, the qualitative in-depth analysis of the present study indicates a relationship between positively reported expected outcomes and patient satisfaction. This is also in line with Kraska et al [27], who demonstrated outcome quality to be a predictor for patient satisfaction during hospital stays in Germany.

Considering the suitability of the applied PCC framework [15], this analysis revealed that the inductively originated domain of “peer relationships” has been of relevance for inpatient rehabilitation programs. As the overall accuracy of the applied taxonomy to the setting of PCC in inpatient rehabilitation was rated high, the “peer relationships” domain may be a meaningful extension of the taxonomy for settings in which patients have the opportunity to interact with their peers over a longer period of time.

In this analysis, patients perceived a significant number of medical staff to present aspects of a developing gratification crisis reflecting generic psychological distress. This finding is supported by Dobson et al [28], who identified health care workers to face moderate levels of depression and anxiety. This observation is particularly prevalent for the nursing profession, which experienced high levels of burnout and emotional exhaustion during the SARS-CoV-2 pandemic [29,30]. To facilitate the resilience of health systems for future pandemic events, it will be of interest to meet health care providers’ needs not only to foster employee health but also to support quality of care. As psychological distress of medical staff was perceived to be an amplifying factor for reduced patient autonomy, it is relevant to refer to the scoping review of Klemmt et al [31] supporting the influence of medical staff on patient autonomy, while further emphasizing domains of the rehabilitation system, the rehabilitation facility, and patients themselves to have a bidirectional influence on autonomy. Following their conclusion, it is important to be aware that IRS not only aims to foster social inclusion as a summative outcome but should also be requested for structures and stakeholders during rehabilitation.

Despite illustrated challenges of IRS during the early phase of the SARS-CoV-2 pandemic, most patients felt safe and supported infection protection standards. This is underpinned by a survey of oncological patients treated in German rehabilitation clinics, 87% of whom reported to feel safe in facilities [32]. Although the implementation of infection-protection standards was associated with a tangible workload increase, 84% of staff members assisted the implementation [32].

### Strengths and Limitations

First, one limitation of our study is the limited representativeness of findings for other rehabilitation settings within Germany as hygiene regulations differed across states. Moreover, online reported patient complaints as a scientific data source produce concerns of representativeness and subjectivity as sample characteristics are uncontrolled and widely unknown. However, an analysis of Facebook reviews demonstrated that contents of reviews do not correlate with inpatient quality assessment indicators but instead correlate with a standardized national survey of patient experiences in German obstetrics [33]. Moreover, a Dutch investigation of online patient ratings identified a positive correlation of these ratings with evaluation reports of the Dutch Healthcare Inspectorate referring to underperforming, high-risk hospitals [34]. Demonstrating initial representativeness concerns of online ratings, Dutch health care inspectors valued online ratings as an additional source of information after being confronted with negative ratings and emphasized to cautiously interpret them under referral to standardized quality and safety indicators [35].

Second, using online reported patient complaints is accompanied by unknown sample characteristics and thereby associated with hazards of selection bias. In this context, Han and colleagues [36] identified prognostic factors of patient characteristics associated with patient intentions and behaviors on physician rating websites. In their survey study, they identified health-related variables (seeking physician information online, usage of web-based medical consultation services, prevalence of a serious disease, good medical experiences) to be directed to the active rating behavior. Conversely, cognitive variables (altruism, self-efficacy to perform online ratings, trust in online ratings of peers) affected the rating intention. These results may help to further understand the patient population using this feedback opportunity.

Moreover, research activities of economic sciences identified online product ratings to be influenced by social dynamics. It is acknowledged that product ratings are not only affected by individual experiences but rather by prior ratings of one’s peer group [37,38]. At this stage of research, it is reasonable to question to what extent these dynamics equally occur on physician and hospital rating websites.

Along with these stated limitations, this analysis faces unique restrictions. As the distribution of reports across included clinics varied strongly, a cluster bias of included rehabilitation clinics with disproportionately strong patient rating activities of some facilities cannot fully be ruled out. In this regard, it will be of interest to further investigate which clinic-related parameters affected ratings of clinics with above-average report numbers. Additionally, the current selection of keywords reflecting PCC domains was made inductively and potentially implies an incomplete list of keywords supporting a distortion of distributed domains.

Despite these limitations, patient rating portals became increasingly popular over the last 10 years [8,39], which suggests that patients claim these portals as a trusted source of information. Beyond a growing number of physicians acknowledging patient reports for in-house quality improvement initiatives [40], by systematically investigating online reported patient reviews, this analysis provides the potential to integrate patient perspectives into the discussion on how to maintain PCC structures under the stress and strains of a pandemic. Integrating online-reported patient complaints offers the opportunity to extend scientific data sources by providing the advantage to reduce the social desirability bias of common qualitative research formats, as this analysis demonstrates that patients perceive a hospital rating website to be a protective platform supporting the exchange of individual experiences.

Taking the present results into account, future research direction should investigate country-specific differences in the perceived significance of PCC domains. For instance, it remains to be answered why online reports of German inpatient care recipients are currently dominated by reports about the care environment, whereas health-relevant outcome expectations seem to have a subordinate role.

### Conclusion

This analysis reflects previous research as German patients predominantly reported feedback associated with the care environment. SARS-CoV-2–related reports were strongly affected by aspects of patient-centered processes addressing discontinuous communication and an organizationally demanding implementation of infection protection standards, which was in some cases perceived to threaten patient autonomy. This perceived threat in reduced autonomy was amplified by a tangible increase in staff workload. Developed peer relationships during the rehabilitation stay had the potential to mitigate these mechanisms.

## Appendix

Multimedia Appendix 1 Data extraction code for web scraping.

Multimedia Appendix 2 COREQ (Consolidated criteria for Reporting Qualitative research) checklist.

Multimedia Appendix 3 Synopsis online reports (original German Version).

## Abbreviations

COREQ: consolidated criteria for reporting qualitative research
IRS: inpatient rehabilitation service
PCC: patient-centered care

## Data Availability

The open-access data are available on Klinikbewertungen [20]. Extracted raw data of included rehabilitation clinics can also be requested from the corresponding author (LK). A synopsis of key statements (German version) can be accessed via Multimedia Appendix 3.
