# Supplementary material for: SARS-CoV-2–Related Adaptation Mechanisms of Rehabilitation Clinics Affecting Patient-Centered Care: Qualitative Study of Online Patient Reports
Source: JMIR Rehabil Assist Technol. 2023 Apr 13;10:e39512. doi: 10.2196/39512 (PMC10131839; doi:10.2196/39512)
Supplement: Multimedia Appendix 1 [file rehab_v10i1e39512_app1.docx]

Data extraction ([www.klinikbewertungen.de](http://www.klinikbewertungen.de))

library(rvest)

#Hohenelse KLinik

page_hohenelse <- read_html("https://www.klinikbewertungen.de/klinik-forum/erfahrung-mit-klinik-hohenelse-der-drv/bewertungen?allbew#more")

inhalte = page_hohenelse %>% html_nodes(".bewertung") %>% html_nodes(".report p") %>% html_text()

diagnose = page_hohenelse %>% html_nodes(".report dd:nth-child(6)") %>% html_text2()

datum = page_hohenelse %>% html_nodes("time") %>% html_text()

View(inhalte)

View(diagnose)

View(datum)

View(diagnose)

hohenelse_2 = data.frame(datum, inhalte, stringsAsFactors = FALSE)

View(hohenelse_2)

library(openxlsx)

write.xlsx(hohenelse_2, "hohenelse_2.xlsx")

#Brandenburg Klinik Bernau-Waldsiedlung

page_brandenburgklinik <- read_html("https://www.klinikbewertungen.de/klinik-forum/erfahrung-mit-brandenburgklinik-bernau/bewertungen?allbew#more")

datum_brandenburgklinik = page_brandenburgklinik %>% html_nodes("time") %>% html_text()

inhalte_brandenburglinik = page_brandenburgklinik %>% html_nodes(".bewertung") %>% html_nodes(".report p") %>% html_text()

View(inhalte_brandenburglinik)

View(datum_brandenburgklinik)

brandenburgklinik = data.frame(datum_brandenburgklinik, inhalte_brandenburglinik, stringsAsFactors = FALSE)

View(brandenburgklinik)

write.xlsx(brandenburgklinik, "brandenburgklinik_bernau.xlsx")

#Fachklinik und Moorbad Bad Freienwalde

page_moorbadklinik <- read_html("https://www.klinikbewertungen.de/klinik-forum/erfahrung-mit-fachklinik-bad-freienwalde-reha-klinik/bewertungen?allbew#more")

datum_moorbadklinik = page_moorbadklinik %>% html_nodes("time") %>% html_text()

inhalte_moorbadklinik = page_moorbadklinik %>% html_nodes(".bewertung") %>% html_nodes(".report p") %>% html_text()

moorbadklinik = data.frame(datum_moorbadklinik, inhalte_moorbadklinik, stringsAsFactors = FALSE)

write.xlsx(moorbadklinik, "moorbadklinik_bad_freienwalde.xlsx")

#Fontane-Klinik Mittenwalde

page_fontaneklinik <- read_html("https://www.klinikbewertungen.de/klinik-forum/erfahrung-mit-fontane-klinik-motzen-mittenwalde/bewertungen?allbew#more")

datum_fontaneklinik = page_fontaneklinik %>% html_nodes("time") %>% html_text()

inhalte_fontaneklinik = page_fontaneklinik %>% html_nodes(".bewertung") %>% html_nodes(".report p") %>% html_text()

fontaneklinik = data.frame(datum_fontaneklinik, inhalte_fontaneklinik, stringsAsFactors = FALSE)

write.xlsx(fontaneklinik, "fontaneklinik_mittenwalde.xlsx")

#GLGFachklinik Wolletzsee Angermünde

page_FachklinikWolletzsee <- read_html("https://www.klinikbewertungen.de/klinik-forum/erfahrung-mit-fachklinik-am-wolletzsee/bewertungen?allbew#more")

datum_FachklinikWolletzsee = page_FachklinikWolletzsee %>% html_nodes("time") %>% html_text()

inhalte_FachklinikWolletzsee = page_FachklinikWolletzsee %>% html_nodes(".bewertung") %>% html_nodes(".report p") %>% html_text()

fachklinikwolletzsee = data.frame(datum_FachklinikWolletzsee, inhalte_FachklinikWolletzsee, stringsAsFactors = FALSE)

write.xlsx(fachklinikwolletzsee, "Fachklinik_Wolletzsee.xlsx")

# Heinrich Heine Klinik Potsdam

page_heinrichheineklinik <- read_html("https://www.klinikbewertungen.de/klinik-forum/erfahrung-mit-heinrich-heine-klinik/bewertungen?allbew#more")

datum_heinrichheineklinik = page_heinrichheineklinik %>% html_nodes("time") %>% html_text()

inhalte_heinrichheineklinik = page_heinrichheineklinik %>% html_nodes(".bewertung") %>% html_nodes(".report p") %>% html_text()

heinrichheineklinik = data.frame(datum_heinrichheineklinik, inhalte_heinrichheineklinik, stringsAsFactors = FALSE)

write.xlsx(heinrichheineklinik, "Heinrich_Heine_Klinik_Potsdam.xlsx")

#Heliosklinik Hohenstücken Brandenburg an der Havel

page_hohenstuecken <- read_html("https://www.klinikbewertungen.de/klinik-forum/erfahrung-mit-helios-hohenstuecken")

datum_hohenstuecken = page_hohenstuecken %>% html_nodes("time") %>% html_text()

inhalte_hohenstuecken = page_hohenstuecken %>% html_nodes(".bewertung") %>% html_nodes(".report p") %>% html_text()

hohenstuecken = data.frame(datum_hohenstuecken, inhalte_hohenstuecken, stringsAsFactors = FALSE)

write.xlsx(hohenstuecken, "Heliosklinik_Hohenstücken.xlsx")

#Klinik am See Rüdersdorf bei Berlin

page_klinikamsee <- read_html("https://www.klinikbewertungen.de/klinik-forum/erfahrung-mit-klinik-am-see-ruedersdorf/bewertungen?allbew#more")

datum_klinikamsee = page_klinikamsee %>% html_nodes("time") %>% html_text()

inhalte_klinikamsee = page_klinikamsee %>% html_nodes(".bewertung") %>% html_nodes(".report p") %>% html_text()

klinikamsee = data.frame(datum_klinikamsee, inhalte_klinikamsee, stringsAsFactors = FALSE)

write.xlsx(klinikamsee, "klinik_am_see_rüdersdorf.xlsx")

#Kliniken Beelitz GmbH

page_klinikenbeelitz <- read_html("https://www.klinikbewertungen.de/klinik-forum/erfahrung-mit-neurologische-reha-heilstaetten/bewertungen?allbew#more")

datum_klinikenbeelitz = page_klinikenbeelitz %>% html_nodes("time") %>% html_text()

inhalte_klinikenbeelitz = page_klinikenbeelitz %>% html_nodes(".bewertung") %>% html_nodes(".report p") %>% html_text()

klinikenbeelitz = data.frame(datum_klinikenbeelitz, inhalte_klinikenbeelitz, stringsAsFactors = FALSE)

write.xlsx(klinikenbeelitz, "Neuro_Reha_beelitz.xlsx")

#Elbtalklinik Bad Wilsnack

page_elbtalklinik <- read_html("https://www.klinikbewertungen.de/klinik-forum/erfahrung-mit-kmg-elbtalklinik-bad-wilsnack/bewertungen?allbew#more")

datum_elbtalklinik = page_elbtalklinik %>% html_nodes("time") %>% html_text()

inhalte_elbtalklinik = page_elbtalklinik %>% html_nodes(".bewertung") %>% html_nodes(".report p") %>% html_text()

elbtalklinik = data.frame(datum_elbtalklinik, inhalte_elbtalklinik, stringsAsFactors = FALSE)

write.xlsx(elbtalklinik, "Elbtalklinik_Bad_Wilsnack.xlsx")

# Mutter-Kind-Klinik Waldfrieden in Buckow

page_waldfrieden <- read_html("https://www.klinikbewertungen.de/klinik-forum/erfahrung-mit-mutter-kind-klinik-waldfrieden-buckow/bewertungen?allbew#more")

datum_waldfrieden = page_waldfrieden %>% html_nodes("time") %>% html_text()

inhalte_waldfrieden = page_waldfrieden %>% html_nodes(".bewertung") %>% html_nodes(".report p") %>% html_text()

waldfrieden = data.frame(datum_waldfrieden, inhalte_waldfrieden, stringsAsFactors = FALSE)

write.xlsx(waldfrieden, "Mutter_Kind_Klinik_Waldfrieden_Buckow.xlsx")

#Median Klinik Grünheide

page_mediangruenheide <- read_html("https://www.klinikbewertungen.de/klinik-forum/erfahrung-mit-median-klinik-gruenheide/bewertungen?allbew#more")

datum_mediangruenheide = page_mediangruenheide %>% html_nodes("time") %>% html_text()

inhalte_mediangruenheide = page_mediangruenheide %>% html_nodes(".bewertung") %>% html_nodes(".report p") %>% html_text()

mediangruenheide = data.frame(datum_mediangruenheide, inhalte_mediangruenheide, stringsAsFactors = FALSE)

write.xlsx(mediangruenheide, "Medianklinik_Grünheide.xlsx")

#Median Klinik Hoppegarten

page_medianhoppegarten <- read_html("https://www.klinikbewertungen.de/klinik-forum/erfahrung-mit-median-klinik-hoppegarten/bewertungen?allbew#more")

datum_medianhoppegarten = page_medianhoppegarten %>% html_nodes("time") %>% html_text()

inhalte_medianhoppegarten = page_medianhoppegarten %>% html_nodes(".bewertung") %>% html_nodes(".report p") %>% html_text()

medianhoppgarten = data.frame(datum_medianhoppegarten, inhalte_medianhoppegarten, stringsAsFactors = FALSE)

write.xlsx(medianhoppgarten, "Median_klinik_Hoppegarten.xlsx")

#MediClin Rehazentrum Spreewald (Burg)

page_mediclin <- read_html("https://www.klinikbewertungen.de/klinik-forum/erfahrung-mit-reha-zentrum-spreewald-brandenburg/bewertungen?allbew#more")

datum_mediclin = page_mediclin %>% html_nodes("time") %>% html_text()

inhalte_mediclin = page_mediclin %>% html_nodes(".bewertung") %>% html_nodes(".report p") %>% html_text()

mediclin = data.frame(datum_mediclin, inhalte_mediclin, stringsAsFactors = FALSE)

write.xlsx(mediclin, "Mediclin_Spreewald.xlsx")

#Rehaklinik Hoher Fläming Bad Belzig

page_hoherflaeming <- read_html("https://www.klinikbewertungen.de/klinik-forum/erfahrung-mit-reha-klinikum-hoher-flaming-belzig/bewertungen?allbew#more")

datum_hoherflaeming = page_hoherflaeming %>% html_nodes("time") %>% html_text()

inhalte_hoherflaeming = page_hoherflaeming %>% html_nodes(".bewertung") %>% html_nodes(".report p") %>% html_text()

hoherflaeming = data.frame(datum_hoherflaeming, inhalte_hoherflaeming, stringsAsFactors = FALSE)

write.xlsx(hoherflaeming, "Rehaklinik_Hoher_Fläming.xlsx")

#Sana Klinik Sommerfeld Kremmen

page_sommerfeld <- read_html("https://www.klinikbewertungen.de/klinik-forum/erfahrung-mit-sana-kliniken-sommerfeld--kremmen/bewertungen?allbew#more")

datum_sommerfeld = page_sommerfeld %>% html_nodes("time") %>% html_text()

inhalte_sommerfeld = page_sommerfeld %>% html_nodes(".bewertung") %>% html_nodes(".report p") %>% html_text()

sommerfeld = data.frame(datum_sommerfeld, inhalte_sommerfeld, stringsAsFactors = FALSE)

write.xlsx(sommerfeld, "Sommerfeld_Kremmen.xlsx")

#Rehazentrum Lübben

page_rehalübben <- read_html("https://www.klinikbewertungen.de/klinik-forum/erfahrung-mit-reha-zentrum-luebben/bewertungen?allbew#more")

datum_rehaluebben = page_rehalübben %>% html_nodes("time") %>% html_text()

inhalte_rehaluebben = page_rehalübben %>% html_nodes(".bewertung") %>% html_nodes(".report p") %>% html_text()

rehaluebben = data.frame(datum_rehaluebben, inhalte_rehaluebben, stringsAsFactors = FALSE)

write.xlsx(rehaluebben, "Rehazentrum_Lübben.xlsx")

#Rehazentrum Seehof (DRV) Teltow

page_drvteltow <- read_html("https://www.klinikbewertungen.de/klinik-forum/erfahrung-mit-reha-zentrum-seehof/bewertungen?allbew#more")

datum_drvteltow = page_drvteltow %>% html_nodes("time") %>% html_text()

inhalte_drvteltow = page_drvteltow %>% html_nodes(".bewertung") %>% html_nodes(".report p") %>% html_text()

drvteltow = data.frame(datum_drvteltow, inhalte_drvteltow, stringsAsFactors = FALSE)

write.xlsx(drvteltow, "Rehazentrum_Teltow.xlsx")

#Salusklinik Lindow

page_salusklinik <- read_html("https://www.klinikbewertungen.de/klinik-forum/erfahrung-mit-salus-klinik-lindow/bewertungen?allbew#more")

datum_salusklinik = page_salusklinik %>% html_nodes("time") %>% html_text()

inhalte_salusklinik = page_salusklinik %>% html_nodes(".bewertung") %>% html_nodes(".report p") %>% html_text()

salusklinik = data.frame(datum_salusklinik, inhalte_salusklinik, stringsAsFactors = FALSE)

write.xlsx(salusklinik, "Salusklinik_Kremmen.xlsx")

#Rehabilitationsklinik Märkische Schweiz Buckow

page_maerkische_schweiz <- read_html("https://www.klinikbewertungen.de/klinik-forum/erfahrung-mit-rehaklinik-buckow")

datum_maerikische_schweiz = page_maerkische_schweiz %>% html_nodes("time") %>% html_text()

inhalte_maerkische_schweiz = page_maerkische_schweiz %>% html_nodes(".bewertung") %>% html_nodes (".report p") %>% html_text()

maerkische_schweiz = data.frame(datum_maerikische_schweiz, inhalte_maerkische_schweiz, stringsAsFactors = FALSE)

write.xlsx(maerkische_schweiz, "Rehaklinik_Maerkische_Schweiz.xlsx")

#Psychotherapeutische Klinik Bad Liebenwerda

page_psych_liebenwerda <- read_html("https://www.klinikbewertungen.de/klinik-forum/erfahrung-mit-psychotherapeutische-klinik-bad-liebenwerda")

datum_psych_liebenwerda = page_psych_liebenwerda %>% html_nodes("time") %>% html_text()

inhalte_psych_liebenwerda = page_psych_liebenwerda %>% html_nodes(".bewertung") %>% html_nodes(".report p") %>% html_text()

psych_liebenwerda = data.frame(datum_psych_liebenwerda, inhalte_psych_liebenwerda, stringsAsFactors = FALSE)

write.xlsx(psych_liebenwerda, "Psychotherapeutische_Klinik_Bad_Liebenwerda.xlsx")

#FONTANA Klinik Bad Liebenwerda

page_FONTANA_liebenwerda <- read_html("https://www.klinikbewertungen.de/klinik-forum/erfahrung-mit-fontana-klinik-bad-liebenwerda/bewertungen?allbew#more")

datum_FONTANA_liebenwerda = page_FONTANA_liebenwerda %>% html_nodes("time") %>% html_text()

inhalte_FONTANA_liebenwerda = page_FONTANA_liebenwerda %>% html_nodes(".bewertung") %>% html_nodes(".report p") %>% html_text()

FONTANA_liebenwerda = data.frame(datum_FONTANA_liebenwerda, inhalte_FONTANA_liebenwerda, stringsAsFactors = FALSE)

write.xlsx(FONTANA_liebenwerda, "Fontana_Klinik_Bad_Liebenwerda.xlsx")

#Seeklinik Zechlin

page_zechlin <- read_html("https://www.klinikbewertungen.de/klinik-forum/erfahrung-mit-seeklinik-zechlin-dorf-brandenburg/bewertungen?allbew#more")

datum_zechlin = page_zechlin %>% html_nodes("time") %>% html_text()

inhalte_zechlin = page_zechlin %>% html_nodes(".bewertung") %>% html_nodes(".report p") %>% html_text()

zechlin = data.frame(datum_zechlin, inhalte_zechlin, stringsAsFactors = FALSE)

write.xlsx(zechlin, "Seeklinik_Zechlin.xlsx")

#Rehakliniken Saarland

library(rvest)

#Bosenbergklinik St. Wendel

page_Bosenberglinik <- read_html("https://www.klinikbewertungen.de/klinik-forum/erfahrung-mit-mediclin-bosenberg-kliniken/bewertungen?allbew#more")

datum_bosenberg = page_Bosenberglinik %>% html_nodes("time") %>% html_text()

inhalte_bosenberg = page_Bosenberglinik %>% html_nodes(".bewertung") %>% html_nodes(".report p") %>% html_text()

Bosenberg = data.frame(datum_bosenberg, inhalte_bosenberg, stringsAsFactors = FALSE)

library(openxlsx)

write.xlsx(Bosenberg, "Bosenbergklinik_StWendel.xlsx")

#Mediclin Bliestal Kliniken

page_bliestalklinik <- read_html("https://www.klinikbewertungen.de/klinik-forum/erfahrung-mit-bliestal-kliniken-mediclin/bewertungen?allbew#more")

datum_bliestal = page_bliestalklinik %>% html_nodes("time") %>% html_text()

inhalte_bliestal = page_bliestalklinik %>% html_nodes (".bewertung") %>% html_nodes(".report p") %>% html_text()

Bliestal = data.frame(datum_bliestal, inhalte_bliestal, stringsAsFactors = FALSE)

write.xlsx(Bliestal, "Bliestalklinik_Blieskastel.xlsx")

#Median Klinik Münchwies

page_Münchwiesklinik <- read_html("https://www.klinikbewertungen.de/klinik-forum/erfahrung-mit-median-klinik-muenchwies-neunkirchen/bewertungen?allbew#more")

datum_münchwies = page_Münchwiesklinik %>% html_nodes("time") %>% html_text()

inhalte_münchwies = page_Münchwiesklinik %>% html_nodes(".bewertung") %>% html_nodes(".report p") %>% html_text()

Münchwies = data.frame(datum_münchwies, inhalte_münchwies, stringsAsFactors = FALSE)

write.xlsx (Münchwies, "Medianklinik_Münchwies.xlsx")

#Median Klinik Berus

page_berusklinik <- read_html("https://www.klinikbewertungen.de/klinik-forum/erfahrung-mit-median-klinik-berus-ueberherrn-berus/bewertungen?allbew#more")

datum_berus = page_berusklinik %>% html_nodes("time") %>% html_text()

inhalte_berus = page_berusklinik %>% html_nodes(".bewertung") %>% html_nodes(".report p") %>% html_text()

Berus = data.frame(datum_berus, inhalte_berus, stringsAsFactors = FALSE)

write.xlsx(Berus, "Medianklinik_ÜberherrnBerus.xlsx")

#Hochwaldklinik Weiskirchen

page_hochwaldklinik <- read_html("https://www.klinikbewertungen.de/klinik-forum/erfahrung-mit-hochwald-kliniken-weiskirchen/bewertungen?allbew#more")

datum_hochwald = page_hochwaldklinik %>% html_nodes("time") %>% html_text()

inhalte_hochwald = page_hochwaldklinik %>% html_nodes(".bewertung") %>% html_nodes(".report p") %>% html_text()

Hochwaldklinik = data.frame(datum_hochwald, inhalte_hochwald, stringsAsFactors = FALSE)

write.xlsx(Hochwaldklinik, "Hochwaldklinik_Weiskirchen.xlsx")

#Fachklinik St. Hedwig Illingen

page_hedwig <- read_html("https://www.klinikbewertungen.de/klinik-forum/erfahrung-mit-fachklinik-st-hedwig-saarland-illingen/bewertungen?allbew#more")

datum_hedwig = page_hedwig %>% html_nodes("time") %>% html_text()

inhalte_hedwig = page_hedwig %>% html_nodes(".bewertung") %>% html_nodes(".report p") %>% html_text()

Hedwig = data.frame(datum_hedwig, inhalte_hedwig, stringsAsFactors = FALSE)

write.xlsx(Hedwig, "Fachklinik_StHedwig_Illingen.xlsx")

#Mutter-Kind-Klinik Saarwald Nohfelden

page_saarwald <- read_html("https://www.klinikbewertungen.de/klinik-forum/erfahrung-mit-klinik-saarwald/bewertungen?allbew#more")

datum_saarwald = page_saarwald %>% html_nodes("time") %>% html_text()

inhalte_saarwald = page_saarwald %>% html_nodes(".bewertung") %>% html_nodes(".report p") %>% html_text()

Saarwald = data.frame(datum_saarwald, inhalte_saarwald, stringsAsFactors = FALSE)

write.xlsx(Saarwald, "MutterKindKlinik_Saarwald_Nohfelden.xlsx")

#Johannesbad Fachklinik Mettlach

page_johannesbad <- read_html("https://www.klinikbewertungen.de/klinik-forum/erfahrung-mit-zentrum-saarschleife-mettlach-orscholz/bewertungen?allbew#more")

datum_johannesbad = page_johannesbad %>% html_nodes("time") %>% html_text()

inhalte_johannesbad = page_johannesbad %>% html_nodes(".bewertung") %>% html_nodes(".report p") %>% html_text()

johannesbad = data.frame(datum_johannesbad, inhalte_johannesbad, stringsAsFactors = FALSE)

write.xlsx(johannesbad, "Fachklinik_Johannesbad_Mettlach.xlsx")
