# Supplementary material for: SARS-CoV-2–Related Adaptation Mechanisms of Rehabilitation Clinics Affecting Patient-Centered Care: Qualitative Study of Online Patient Reports
Source: JMIR Rehabil Assist Technol. 2023 Apr 13;10:e39512. doi: 10.2196/39512 (PMC10131839; doi:10.2196/39512)
Supplement: Multimedia Appendix 3 [file rehab_v10i1e39512_app3.docx]

**Kernaussagen patientenzentrierte Versorgung Rehakliniken unter SARS-CoV2 - Synopse**

| **Domain** | **Category** | **Subcategory** | **Kernaussage** | **Zitat** | **Bezug zu SARS-CoV2** |
| --- | --- | --- | --- | --- | --- |
| **Prerequisites** | Health professionals have appropriate skills and knowledge | Professional competence | **mS** |  |  |
|  |  | Attributes of the patient-centred professional | Abrechnung nicht erbrachter Therapieleistungen schwächt Vertrauensverhältnis | *„Nun noch zu meinem Abschlussbericht. Ich weiß gar nicht ob ich dagegen vorgehen soll. Dort steht, ich hätte für mein Wohlbefinden Anwendungen auf der Medijet-Massageliege bekommen. Ich habe keine bekommen weiß gar nicht was das ist.Ich hätte angegeben das ich gut erholt aus der Reha gehe. Das stimmt alles nicht. Dann stehen falsche Daten im Bericht. Ganz viel Unwahrheiten. So etwas hätte ich von Burg nie gedacht.“*  (Mediclin_Spreewald_Burg, Spalte: 2 \| Zeile: 23) | NEIN |
|  |  |  | Gratifikationskrise des medizinischen Personals | *„Wenn man sich beklagt wird sofort zurückgeschossen und man muss das Maul halten und sich zusammenreißen, damit man das nächste Mal überhaupt bedient wird. Auch bei Ärzten und Therapeuten ist mir aufgefallen, dass manche wohl meinen, Patienten seien minderwertig und wären zur Umerziehung hier. Ganz allgemein kommt es mir so vor, als hätten alle die Lust am Job verloren. Sowas kommt meist von Oben und wird in Kauf genommen, solange man Geld sparen kann. GANZ KLAR: es gibt Ausnahmen“*  *(Bliestalklinik_Blieskastel, Spalte: 2 \| Zeile: 5)* | Unklar |
|  | A cohesive and co-operative team of professionals | Cooperation among clinicians a priority | **N/A** |  |  |
|  |  | Differences in perception of role between doctors, nurses, and patients | **N/A** |  |  |
| **Patient-centred processes** | Participation of the patient as a respected and autonomous individual | Respect for patients’ values, preferences, and respected needs | **mS** |  |  |
|  |  | Patient as a source of control | **Diskontinuierliche Kommunikation zu Ausgangsbeschränkungen und Isolation erzeugt Gefühl von Machtlosigkeit** | *„Das schlimmste war aber die Ausgangssperre. Das Klinikgelände durfte ich nicht verlassen, es war nicht möglich einen selbstausgefüllten Bogen zu hinterlegen, auf dem ich hätte dokumentieren können, wann ich das Klinikgelände verlasse und wieder zurückkomme, reine Willkür der Klinik, den in anderen Rehakliniken ist keine Ausgangssperre und dort werden auch Einzelwassertherapien angeboten.“*  (Bliestalklinik_Blieskastel, Spalte: 2 \| Zeile: 25)  *„Willkürliche Ausgangssperren wurden durch die Leitung verhängt und zwei Stunden später nur durch Mund zu Mund Propaganda wieder aufgehoben. Die Leitung stellte sich zu keiner Zeit den Fragen der Patienten sondern versteckte sich. Corona Fälle wurden bei Nacht und Nebel heimlich ausgeschleust. Trotzdem wurde behauptet, dass es keine bestätigten Fälle gäbe. Getestet wurden nur Insassen mit akuten Symptomen.“*  (Bliestalklinik_Blieskastel, Spalte: 2 \| Zeile: 31) | JA |
|  |  | Patient’s active involvement and participation | **N/A** |  |  |
|  |  | Patient autonomy | **Um Hygienevorschriften zu wahren, wird die Entscheidungsautonomie von Patient*innen eingeschränkt 🡪 Gefühl von Willkür** | *„Allgemein entsteht ein Gefängnischarakter durch den entmündigenden, verständnislosen Habitus mancher TherapeutInnen. Wem Selbstbestimmung ein wichtiger Wert ist, der wird hier nicht glücklich werden.“*  (Medianklinik_Münchwies, Spalte: 2 \| Zeile: 2)  *„Nie wieder [Klinik XY] Stern ist Zuviel, Erwachsene Menschen müßten hier Duldungsgeld erhalten. Nach insgesamt 2 1/2 Wochen Aufenthalt in diesem Knast bin ich froh wieder zu Hause sein zu dürfen. Ich wäre auch keinen Tag länger dortgeblieben. Als Haupt Aufenthaltsraum dient die Eingangshalle (gut gelüftet und saukalt), bewacht von diversen Damen (die intern Rottweiler oder Gruppenführer) genannt wurden. Maske schief gab anpfiff. Vor der Tür treffen mit Angehörigen mit 4 Meter Abstand wurde sofort unterbunden, Pizzafahrer in [Ort] sind offensichtlich Corona immun, denn diese durften Hausgäste draußen empfangen und ein Schwätzchen halten. […] Im Fütterungssaal hingegen wurde eiserne Disziplin gefordert, Abstand Maske und an die Disziplin in der Reihe „*  (Fachklinik_Johannesbad_Mettlach, Spalte: 2 \| Zeile: 2)  *„Willkürliche Ausgangssperren wurden durch die Leitung verhängt und zwei Stunden später nur durch Mund zu Mund Propaganda wieder aufgehoben. Die Leitung stellte sich zu keiner Zeit den Fragen der Patienten sondern versteckte sich. Corona Fälle wurden bei Nacht und Nebel heimlich ausgeschleust. Trotzdem wurde behauptet, dass es keine bestätigten Fälle gäbe. Getestet wurden nur Insassen mit akuten Symptomen.“*  (Bliestalklinik_Blieskastel, Spalte: 2 \| Zeile: 31) | JA |
|  | Involvement of family and friends | Family and friends supported as caregivers | Mangelndes Freizeitangebot für Begleitpersonen | “*Als Eltern hängt man ausschließlich rum, was sehr frustriert.”* (Heliosklinik_Hohenstücken, Spalte: 2 \| Zeile: 4) | Unklar |
|  | Care plan based on patient’s individual needs | Care customized according to patient needs and values | Behandlungsplan nicht auf Patientenbedürfnisse abgestimmt | *„Coronabedingt erstmal Quarantäne, ok, Therapien sehr weit gestreut, ich frage mich immer noch, was Wechselbäder Ellbogen/Füsse mit meinem Krankheitsbild zu tun haben, dafür hatte ich diese jeden 2.Tag.“*  (Mediclin_Spreewald_Burg, Spalte: 2 \| Zeile: 12) | JA |
|  |  |  | Individualisierte Therapiepläne werden trotz Kurzarbeit gewährleistet | *„Obwohl viele Mitarbeiter der Klinik in Kurzarbeit sind, hatte ich doch einen gut gefüllten Therapieplan, der mir viel Freude und auch Muskelkater bescherte. Alle Therapeuten geben sich große Mühe, und nehmen viel Rücksicht auf Patienten, die nicht so können wie Sie möchten.“* (Rehaklinik_Maerkische_Schweiz, Spalte: 2 \| Zeile: 2) | JA |
|  |  | Needs are anticipated | **N/A** |  |  |
|  |  | Coordination and integration of care | **mS** |  |  |
|  |  | Transition and continuity of care | Sektorenübergreifende Versorgungskontinuität wird nicht aufrecht erhalten | *„Die Betreuung während meiner Reha war einwandfrei, aber die "Nachsorge" lässt sehr zu wünschen übrig. Leider ist die Sozialarbeiterin nicht erreichbar, trotz mehrfacher Anrufe, Rückrufbitten und Anfragen per Mail bzgl. meiner Wiedereingliederung und fehlender Unterlagen. Hier habe ich festgestellt, dass es mehreren ehemaligen Patienten so erging.“* (Heinrich_Heine_Klinik_Potsdam, Spalte: 2 \| Zeile: 40)  *„Ich war im Mai 2017 kurz nach einem Antrag auf Erwerbsminderungsrente in Lindow zur Reha. Die auf Wunsch mitgebrachten medizinischen Unterlagen wurden nicht mit einbezogen, ebenso mein Psychotherapeut nicht.“* (Salusklinik_Lindow, Spalte: 2 \| Zeile: 7) | Unklar |
|  | Genuince clinician-patient relationship | Care based on a continuous healing relationship | **Angehörige der Pflege- und Therapieberufe werden eher als Vertrauenspersonen gesehen als das ärztliche Personal** | *„Nun die medizinische Seite; die Ärzte, ich sah nur einen jungen syrischen Assistenzarzt, machten Ihren Job mehr recht als schlecht. Herausragend freundlich und kompetent waren die Schwestern und Pflegekräfte, hier kann ich nur in höchsten Tönen loben. Das gilt auch für die Physiotherapeuten und Fitness Betreuer. Da könnte sich die Direktion dieses Hauses (die niemand zu Gesicht bekam) mal was absehen.“*  (Fachklinik_Johannesbad_Mettlach, Spalte: 2 \| Zeile: 2) | NEIN |
|  |  | Clinician-patient relationship | Funktionierende Kliniker-Patienten-Beziehung hat das Potential, unerwünschte Auswirkungen der SARS-CoV2 Pandemie auf den Reha-Aufenthalt abzufedern | *„Ich war für 4 Wochen wegen HWS und Diabetes zur Reha in Hohenelse. Schon die 12 stündige Quarantäne (es war Corona Zeit) verlief wie im Fluge, da sich auch in dieser Zeit von den netten Mitarbeitern und Schwestern jeder um mein Wohl bemühte.“* (hohenelse_Rheinsberg, Spalte: 2 \| Zeile: 8) | JA |
|  | Open communication of knowledge, personal expertise and clinical expertise between the patient and the professional | Knowledge shared and information flows freely | Rassistische Tendenzen der Patient*innen gegenüber des Klinikpersonals | *„Beschwerden über ausländische Mitarbeiter sind unfair und unangebracht. Sie arbeiten sehr gut und helfen aus, wenn Not am Mann ist. Die Verständigung ist wegen der Maskenpflicht oft problematisch.“*  (Seeklinik_Zechlin, Spalte: 2 \| Zeile: 1) | NEIN |
|  |  | Information, communication and education | Wertfreie Kommunikation stärkt Kliniker-Patienten-Beziehung | *„Besonders bedanken möchte ich mich bei dem Klinikleiter, den einen Tag wo mein einer Zwilling wegen der Maske einen ganz doofen Moment hatte. Es fühlte sich soooo gut für mich an das Sie so ruhig und urteilsfrei waren, ich glaube das war das erste Mal überhaupt das mir das Verhalten meines Kindes mal nicht peinlich war. Einfach weil Sie uns das Gefühl gegeben haben das es völlig ok ist, wenn er jetzt mal so ist wie er eben ist. Sie und ihr ganzes Klinikteam haben ein fettes Bienchen verdient :)Also, jede Mama die eine sehr gute Klinik sucht, ab nach [Ort].“* (Mutter_Kind_Klinik_Waldfrieden_Buckow, Spalte: 2 \| Zeile: 20) | NEIN |
|  |  |  | Vermittlung von Gesundheitskompetenz bei chronischen Schmerzpatienten scheitert | *„Es wird sich nicht um die SCHMERZEN gekümmert,im Gegenteil, es wird auf die Psyche oder das Gewicht geschoben. Keine kompetenten Ärzte vorhanden und ständiger Wechsel. Meine quälenden Nervenschmerzen, weswegen ich gekommen bin, wurden nicht behandelt. Habe es vorgezogen nach 11 Tagen nach Hause zu gehen, um mich dort vernünftig behandeln zu lassen. Sorry, aber diese Klinik hat den Namen Schmerztherapie nicht verdient.“ (Sommerfeld_Kremmen, Spalte: 2 \| Zeile: 17)* | Nein |
|  |  | Feedback mechanisms to measure patient experience | **N/A** |  |  |
| **The care environment** | System issues | Geographic accessibility | **N/A** |  |  |
|  |  | Availability | **Verfügbarkeit therapeutischer und pflegerischer Angebote teilweise stark eingeschränkt** | *„In den gut 3 Monaten konnte ich nur 3 mal geduscht werden. Wenn ich mal gefragt habe wie es wäre das ich als inkompletter Querschnittsgelähmter Duschen könnte, wurde mir gesagt zu wenig Personal vielleicht morgen. HÄ wie jetzt. Naja und ganz zuschweigen von dem eingewachsenen Fußnagel. Schmerz. Da wurde mir gesagt die Medizinischefußpflege, ist nicht, wegen Corona weil die von außerhalb kommt. Naja Schmerz lässt auch irgendwann nach...Auch nach Ansprache der Ärzte das ich mich Untertherapiert fühle hat sich nicht wirklich was geändert. Alles in allem eine durchwachsende Meinung und ganz wichtig nicht immer alles auf Corona schieben. Wo ein Wille da auch ein Weg..“* (Medianklinik_Grünheide, Spalte: 2 \| Zeile: 9)  *„Die Therapie wurde von 60min. Dauer, auf 30min. gekürzt, wovon 15min. für Auf-/Abbau sowie Erwärmung benötigt werden.) Um Reittherapie zu bekommen musste ich ganz schön argumentieren, obwohl diese Therapie einer der Hauptgründe zur Wahl dieser Klinik war. 3x20min. auf einem Pferd sitzen in insgesamt 3 Wochen, war es dann auch schon. Ergo-Einzeltherapie fand insgesamt 3 Stunden statt, was viel zu wenig ist - trotz vorheriger Zielsetzung mit Ärzten/Therapeuten. Immer wieder hört man, zu wenig Personal wg. Urlaubszeit. Sorry, das interessiert mich nicht. Die Klinik hatte den Rehazeitraum vorgeschlagen! 1,5 Stunden Therapie am Tag rechtfertigen m.E. dann nicht einen so teuren medizinischen Einsatz über 3 Wochen. Nicht für die Krankenkasse und auch nicht für mich, die jeden Tag 10€ bezahlen + auf Gehalt verzichten muss.“*  (Heliosklinik_Hohenstücken, Spalte: 2 \| Zeile: 4) | JA |
|  |  |  | **Eingeschränktes Freizeitangebot** | *„Coronabedingt gab es keine Angebote für Veranstaltungen. Hier müßte es in dieser Zeit ein Angebot zum Basteln und Töpfern geben.“* (Rehazentrum_Lübben, Spalte: 2 \| Zeile: 11*)*  *„In Coronazeiten keine Freizeitaktivitäten möglich, Schwimmhalle gesperrt, kein Cafe oder ähnliches -> man hatte nur Fernsehen oder lesen, andere nutzten leider die Zeit zum Rauchen und Alkohol trinken!“ (Brandenburg\brandenburgklinik_bernau: 2\|19 - 2\|19)*  *„Die Freizeitmöglichkeiten sind trotz Corona sehr gut und ohne Corona könnte man sich wahrscheinlich gar nicht entscheiden, was man machen soll.“ (Brandenburg\fontaneklinik_mittenwalde: 2\|6 - 2\|6)* | JA |
|  |  | Financial accessibility | **N/A** |  |  |
|  |  | Supportive organizational system | **Gängige Höflichkeitsformen werden unter SARS-CoV2-Bedingungen verstärkt wertgeschätzt** | *„Ein großes Lob an die Küche und das Servicepersonal. Trotz Corona - immer freundlich, immer schnell. Und das Essen war wirklich lecker - vielen Dank! Ich würde mich sehr freuen, wenn ich nochmal wieder kommen darf. Aber bitte nicht wieder wegen einer onkologischen Erkrankung :-)“*  (Rehazentrum_Lübben, Spalte: 2 \| Zeile: 13) | JA |
|  |  |  | **Akzeptanz von Hygieneregeln, wenn diese sich gut in den organisatorischen Ablauf integrieren lassen** | *„Das Personal, angefangen bei den Therapeuten bis zu den Ärzten sind durch die Bank freundlich und kompetent. Die Organisation ist klasse, so werden die erforderlichen Corona-Maßnahmen eng mit dem Gesundheitsamt regelmäßig abgestimmt, angepasst und, was nicht selbstverständlich ist, auch auf Einhaltung überwacht.“* (Bosenbergklinik_StWendel, Spalte: 2 \| Zeile: 3) | JA |
|  |  | Therapeutic environment | Veränderte Abläufe erzeugen persönlicheres, vertrautes Umfeld | *„Auf Grund der Corona- Pandemie wurden Abläufe geändert, was aber der Therapie durchaus auch gut tat. So habe ich die Halbierung der Bezugsgruppe, deren zeitlicher Rahmen auch halbiert wurde, eigentlich als sehr angenehm und persönlicher empfunden. Die Sitzungen empfand ich intensiver und individueller. Hier sollte vielleicht überlegt werden, ob dies nach der Pandemie beibehalten werden kann.“*  (Heinrich_Heine_Klinik_Potsdam, Spalte: 2 \| Zeile: 8) | JA |
|  |  |  | **Ernährungslehre und gelebte Praxis sind inkonsistent** | *„Mich hat nur erstaunt, dass die Vorträge bzgl. kalorienhaltiger Nahrung so gar nicht mit dem Angebot übereingestimmt hat“* (Heinrich_Heine_Klinik_Potsdam, Spalte: 2 \| Zeile: 23)  *„Liebe Klinik....Du hast schon ein paar Jahre auf dem Buckel und das sieht man. Ich hab die Wochen mit Humor genommen und bin froh wieder auf Arbeit zu sein,umgeben von frischem Gemüse und Obst und guten Lebensmitteln.“* (Rehaklinik_Maerkische_Schweiz, Spalte: 2 \| Zeile: 4) | Nein |
|  |  |  | Kliniken mit Hotelcharakter steigern emotionales Wohlbefinden der Patient*innen | *„Ich war zum zweiten Mal im Rehazentrum Lübben nach einer onkologischen Erkrankung. Ich habe mich genau wie beim ersten Mal sofort sehr nett aufgehoben gefühlt. optisch wirkt die Klinik eher wie ein Hotel, was mir ein gutes Gefühl gab. Sie ist hell, lichtdurchflutet und überall lädt es zum Verweilen ein“*  (Rehazentrum_Lübben, Spalte: 2 \| Zeile: 13) | Nein |
| **Expected outcomes** | Adressing a patient’s physical and emotional needs | Physical comfort | Klare Kommunikation von therapierelevanten Endpunkten und deren Veränderung erzeugt Patientenzufriedenheit | *„Nach der Bearbeitung meiner HWS lässt sich mein Hals wie bei einer Eule drehen - wiederum Juhu Da mein "lieber" Doc mich auch gleich auf 1800 Kalorien gesetzt hat, kam noch ein weiterer Erfolg hinzu: 8,8 Kilo und 7,6cm Bauchumfang habe ich in Hohenelse gelassen.“* (hohenelse_Rheinsberg, Spalte: 2 \| Zeile: 8)  *„Ich bin als Diabetiker mit Übergewicht angekommen, habe seit drei Jahren Medikamente u.a. auch gegen Bluthochdruck genommen. Die ganzheitliche Fürsorge des Personals hat dazu geführt, dass ich in vier Wochen 12 Kilogramm abgenommen und nun medikamentenfrei bin. Mein heutiger Langzeitblutzuckerwert liegt bei 5,9 und ich komme von über 8.“* (hohenelse_Rheinsberg, Spalte: 2 \| Zeile: 17)  *„Die Betreuung durch die Ärzte und die Therapeuten kann ich nur loben. Ich kam mit einer starken Schwellung und Erguss im Knie hier an und verlasse die Reha mit sehr guter Beweglichkeit und Stabilität im Gelenk (70° bei Anreise 115° bei Abreise). Die therapeutische Behandlung und Beratung bei den Einzeltherapien ist dabei besonders gut gewesen. Das Fachpersonal ist wirklich kompetent, wenn man gewillt ist sich der Behandlung zu stellen und mit zu arbeiten.“* (Mediclin_Spreewald_Burg, Spalte: 2 \| Zeile: 8) | NEIN |
|  |  | Emotional support-alleviation of anxiety | Emotionale Unterstützung im Umgang mit persönlicher Krankheitsgeschichte fördert Heilungsprozess/Selbstmanagement | *„Neben den mannigfaltigen zusätzlichen Therapieangeboten möchte ich auch besonders die diplompsycholgische Konversation mit Herrn A. hervorheben, dessen Gespräche und umgesetzte Maßnahmen sich überaus positiv auf mein Wohlbefinden auswirkten.“* (Brandenburg\Fachklinik_Wolletzsee: 2\|3 - 2\|3)  *„Ich kann vor dieser Klinik nur warnen. Auf die Patienten wird kaum eingegangen, wodurch sich der gesundheitliche Zustand oft noch verschlechtert.“* (Brandenburg\fontaneklinik_mittenwalde: 2\|2 - 2\|2) | NEIN |
| **Inductive Categories** | Peer relationship | Peer as a supported person of trust | **Funktionierende Peer-Beziehungen haben das Potential, unerwünschte Auswirkungen der SARS-CoV2 Pandemie abzufedern** | *„Ich war für fünf Wochen in der Salus Klinik in Lindow. Die erste Woche war noch sehr geprägt von Unsicherheit vor dem Unbekannten, aber die tollen Menschen die ich vom ersten Tag an kennengelernt habe, unterstützten mich und ich sie, um mit den Problemen die entstanden sind umgehen zu können. Wir haben uns meistens zum Essen verabredet, um uns gegenseitig zu erzählen was der Tag so gebracht hat. Oftmals waren wir auch nur Zuhörer wenn es einem Kollegen von uns schlecht ging und versuchten für ihn da zu sein.“*  (Salusklinik_Lindow, Spalte: 2 \| Zeile: 25) | JA |
|  |  | A call for personal responsibility | **Appell an Eigenverantwortung der Peer Gruppe** | *„Und was geht gar nicht? Wirklich störend empfand ich vor allem das Verhalten einiger MitpatientInnen, die ihre Motzer-Kultur ausleben oder den Raucherpavillon in eine Party-Zone verwandeln, ein Spiegel der Gesellschaft.“* (Medianklinik_ÜberherrnBerus, Spalte: 2 \| Zeile: 4)  *„Eine Rehabilitation ist kein Urlaub, das eigene Mitmachen wird erwartet und ist auch notwendig- der Erfolg hängt maßgeblich von dir und deiner Einstellung zur Reha und zur eigenen Krankheit ab eine Reha-Einrichtung ist kein Hotel mit vielen Sternchen…“* (Heinrich_Heine_Klinik_Potsdam, Spalte: 2 \| Zeile: 14) | NEIN |

**Legende:**

TEXT: Psychiatrie

TEXT: Orthopädie

TEXT: Onkologie

TEXT: Mutter-Kind

TEXT: Neurologie

TEXT: Diabetologie

TEXT: Geriatrie

TEXT: Lymphologie

N/A: Not applicable

mS: Minor significance

JA: Kernaussage hat Bezug zu Bedingungen unter SARS-CoV2 Pandemie

NEIN: Kernaussage hat vermutlich keinen Bezug zu Bedingungen unter SARS-CoV2 Pandemie

Unklar: Unklar, ob Kernaussage Bezug zu Bedingungen unter SARS-CoV2 Pandemie hat

**Fett** markierte Aussagen in Spalte “Kernaussagen“ repräsentieren überdurchschnittliche Häufigkeit im Datenmaterial

Diese Synopse bezieht sich auf online-Rezensionen von Patient*innen mit Aufenthalt in stationären Rehabilitationskliniken (N = 31) der Bundesländer Brandenburg (N = 27, online gelistet N = 23) und Saarland (N = 15, online gelistet N = 8) des Bewertungsportals [www.klinikbewertungen.de](http://www.klinikbewertungen.de) aus dem Zeitraum März 2020 bis September 2021. Eingeschlossen wurden N = 659 Rezensionen (Brandenburg N = 478; range 2-85; Saarland N = 181; range 8-64). Von N = 659 eingeschlossenen Rezensionen wurden N = 10 im genannten Zeitraum verfasst, bezogen sich jedoch auf einen Reha-Aufenthalt vor der SARS-CoV2 Pandemie. Daraus resultieren N = 649 eingeschlossene Rezensionen.
